# Smear plus Detect-TB for a sensitive diagnosis of pulmonary tuberculosis: a cost-effectiveness analysis in an incarcerated population

**DOI:** 10.1186/s12879-014-0678-x

**Published:** 2014-12-16

**Authors:** Karen Barros Schmid, Luciene Scherer, Regina Bones Barcellos, Daniele Kuhleis, Isaías Valente Prestes, Ricardo Ewbank Steffen, Elis Regina Dalla Costa, Maria Lucia Rosa Rossetti

**Affiliations:** Centro de Desenvolvimento Científico e Tecnológico (CDCT), Fundação Estadual de Produção e Pesquisa em Saúde (FEPPS), Av. Ipiranga 5400, 3° andar, Porto Alegre, CEP 90610-000 Rio Grande do Sul, Brazil; Universidade Luterana do Brasil (ULBRA), Canoas, Brazil; Programa Nacional de Controle da Tuberculose - Secretaria de Vigilância em Saúde/Ministério da Saúde, Porto Alegre, Brazil; Programa de Pós-graduação em Epidemiologia da Universidade Federal do Rio Grande do Sul (UFRGS), Porto Alegre, Brazil; Universidade Federal do Rio de Janeiro (UFRJ), Rio de Janeiro, Brazil

**Keywords:** Tuberculosis, Cost-effectiveness, Prison, Detect-TB, Molecular diagnosis, IS6110

## Abstract

**Background:**

Prison conditions can favor the spread of tuberculosis (TB). This study aimed to evaluate in a Brazilian prison: the performance and accuracy of smear, culture and Detect-TB; performance of smear plus culture and smear plus Detect-TB, according to different TB prevalence rates; and the cost-effectiveness of these procedures for pulmonary tuberculosis (PTB) diagnosis.

**Methods:**

This paper describes a cost-effectiveness study. A decision analytic model was developed to estimate the costs and cost-effectiveness of five routine diagnostic procedures for diagnosis of PTB using sputum specimens: a) Smear alone, b) Culture alone, c) Detect-TB alone, d) Smear plus culture and e) Smear plus Detect-TB. The cost-effectiveness ratio of costs were evaluated per correctly diagnosed TB case and all procedures costs were attributed based on the procedure costs adopted by the Brazilian Public Health System.

**Results:**

A total of 294 spontaneous sputum specimens from patients suspected of having TB were analyzed. The sensibility and specificity were calculated to be 47% and 100% for smear; 93% and 100%, for culture; 74% and 95%, for Detect-TB; 96% and 100%, for smear plus culture; and 86% and 95%, for smear plus Detect-TB. The negative and positive predictive values for smear plus Detect-TB, according to different TB prevalence rates, ranged from 83 to 99% and 48 to 96%, respectively. In a cost-effectiveness analysis, smear was both less costly and less effective than the other strategies. Culture and smear plus culture were more effective but more costly than the other strategies. Smear plus Detect-TB was the most cost-effective method.

**Conclusions:**

The Detect-TB evinced to be sensitive and effective for the PTB diagnosis when applied with smear microscopy. Diagnostic methods should be improved to increase TB case detection. To support rational decisions about the implementation of such techniques, cost-effectiveness studies are essential, including in prisons, which are known for health care assessment problems.

**Electronic supplementary material:**

The online version of this article (doi:10.1186/s12879-014-0678-x) contains supplementary material, which is available to authorized users.

## Background

In 2011, 5.8 million newly diagnosed cases were reported to national TB control programs and the World Health Organization (WHO). The prevalence of TB in prisons has been reported to be up to 100 times higher than that of the civilian population [1]. This problem is particularly critical in countries of high and intermediate TB incidence ranging from 0.2% in Europe to 1.9% in Eastern Ethiopia [2]–[7]. Although underestimated, the average incidence of TB in the penitentiary system of southern Brazil in 2006 was 0.7% (725/100,000), 15 times the rate of the general population (48/100,000), according to the State Program of Tuberculosis Control [8]. In a Brazilian study from 2012, the TB incidence was 55/1,900 (2,894/100,000) inhabitants in prisons [7]. Prison conditions can favor disease spread through overcrowding, poor ventilation, malnutrition and the lack of medical care. Additionally, late diagnosis, inadequate treatment and repeated prison transfers promote the transmission of TB infection. Prisons act as a reservoir for TB, carrying the disease into the civilian community through staff, visitors and inadequately treated former inmates [9].

The conventional technique of smear examination with Ziehl-Neelsen (ZN) is widely recognized as inexpensive and easy, but its low sensitivity is a major drawback [10]–[12]. Although the culture technique has proven to be much more sensitive, it is much more time consuming and laborious. Culture, supported by microscopy, still remains the “gold standard” for active TB diagnosis, especially in low-resource countries, where they are the only methods available for confirming TB in patients with a clinical presumption of active disease [13]. Our aim, therefore, is to examine the utility of a molecular method of TB detection within an incarcerated population to find a more rapid alternative that enhances the active search for respiratory symptoms. Many prior studies have observed that the routine clinical use of PCR may be difficult due to its high cost, particularly if PCR is used alone. These studies also emphasize the importance of clinical utility and cost-effectiveness as a basis for making the decision to use this technique [9]–[15].

The Xpert MTB/RIF, a rapid molecular test that can diagnose TB and rifampicin resistance within 100 minutes, is an impressive example of the diagnostic innovations that are currently being implemented. From its endorsement by the WHO in December 2010 to the end of June 2012, 1.1 million tests had been purchased by 67 low- and middle-income countries; South Africa (37% of purchased tests) is the leading adopter. A 41% price reduction (from US$ 16.86 to US$ 9.98) in August 2012 should accelerate uptake [14].

Molecular techniques have the potential to improve clinical care by dramatically reducing the time required for detection and may provide substantial savings in the overall costs of patient care [15]–[17]. Studies using molecular techniques can be performed to evaluate the impact of costs and the cost-effectiveness of new technologies on the health care system. These studies have shown the potential of molecular tests to detect and control TB [18]–[22]. Eight billion dollars per year are necessary for TB control in low- and middle-income countries, resulting in a high impact on the health care system budget [14]. Recently, a new molecular commercial kit has been developed in Brazil for rapid and sensitive pulmonary TB (PTB) diagnosis, Detect-TB (Labtest, MG, Brazil). Detect-TB is based on colorimetric detection of amplified product of the IS*6110* region hybridized with specific probe fixed on microplate [23]. Previous study has shown sensitivity ranging from 75 to 100%, and specificity from 98 to 100% [23].

Considering the innovations in diagnostics and the paucity of reports on health care cost impacts from TB, especially with respect to incarcerated populations, this is the first Brazilian study conducted on this subject. Using spontaneous sputum samples, the following parameters were evaluated: a) The performance and accuracy of the smear, culture and Detect-TB strategies; b) The performance of smear plus culture and smear plus Detect-TB strategies, according to different TB prevalence rates; and c) The cost-effectiveness of routine diagnostic procedures for PTB diagnosis using sputum specimens.

## Methods

### Study desing

This is a cost-effectiveness study. The model A hypothetical cohort of 1000 patients was defined by patients, which were infected with HIV or not, admitted to a Prison in the south region of Brazil.

### Study location and population

All sputum specimens used in this study were obtained from the incarcerated population of the Penitenciária Estadual do Jacuí of Charqueadas, Rio Grande do Sul, Brazil. All samples were obtained from another study performed by our group, in which the TB prevalence was 72/1,900 (4,960/100,000) [7].

### Sample collection

From August 2007 to August 2008, the medical records of patients were admitted to the ambulatory by a screening questionnaire given to 1,900 prisoners [7]. A total of 390 specimens of spontaneous sputum from patients suspected of having TB were eligible for inclusion in the present study. The patients were attended in a clinic room, with a specific area for the collection of this type of material. All sputum samples were taken to the Centro de Desenvolvimento Científico e Tecnológico (CDCT) of the Fundação Estadual de Produção e Pesquisa em Saúde (FEPPS) for processing.

The inclusion criteria were: cough for more than three weeks; a signed informed consent form; and completion of a questionnaire. Each patient was included in the study only once and contributed only one isolate.

Eligible medical records were those in which: a) Patients were confirmed as having PTB and that were clinically notified by SINAM (Information System on Diseases of Compulsory Declaration in Brazil); b) Age > 18 years; and c) Period of hospital stay of more than 1 week. The medical records of patients who were discharged in less than one week from the hospital were excluded from the study.

The medical records of patients with PTB admitted to ambulatory were analyzed. Pulmonary tuberculosis patients carried out 4 smear tests, 4 chest radiographs, and nursing and physician consultations during hospitalization; we used these parameters to estimate the costs of inpatient assistance in the ambulatory, following the Brazilian recommendations for treatment [24].

Chest radiographs and physical examination was performed by a respiratory specialist using a standardized protocol.

### Routine laboratory process

#### Microbiological testing

All PTB suspects provided a single specimen. The samples (500 μL) were treated with 2% N-acetyl-L-cysteine (500 μL)/1 M NaOH (500 μL). The specimens were tested by the Ziehl-Neelsen method (ZN) using smear microscopy, cultured in Löwenstein Jensen and identified according to previous descriptions [24]. Laboratory and tested using the Ziehl- Neelsen method, by culturing in Lowentein Jensen medium with identification performed according to Kubica’s method and employing the following criteria for reading and interpreting: a) No bacillus in 100 fields report result as negative; b) 1 to 10 bacillus in 100 fields report result as the specific quantity found; c) 10 to 99 bacillus in 100 fields report result as positive (+); d) 1 to 10 bacillus in the first 50 fields report result as positive (++); and e) More than 10 bacillus in the first 20 fields report result as positive (+++) [24]. The clinical outcome was chosen as the gold standard.

#### Molecular testing

Laboratory technicians who conducted the molecular testing were blinded to the bacteriology results. DNA extraction, purification and amplification were performed as described previously [23]. To prevent DNA contamination, strict room separation was used, including a work flow from initiation of the PCR to hybridization.

Briefly, Mycobacterium tuberculosis (MTB)-complex DNA was amplified using biotinylated primers targeting the IS6110 fragment. These amplified products were reverse-hybridized on microwell plates (Nunc Immobilizer™ Amino Surface, Nunc A/S, Roskilde, Denmark) to a fixed aminated probe complementary to the internal region of the amplified IS6110 fragment. The hybridization signal was detected by colorimetry using the streptavidin-peroxidase/TMB system and measured using a spectophotometer with a 450/620 nm filter. The absorbance of the negative control was subtracted from the results, as recommended by Detect-TB (Nunc Tech Note, 1999a, Nunc Tech Note, 1999b). All samples with readings above 0.275 were considered positive for MTB complex DNA [23].

#### Ethics

This study was approved by the Ethical Committee of Fundação Estadual de Produção e Pesquisa em Saúde of Rio Grande do Sul (FEPPS-RS) (410528/2006-4).

#### TB case definition

Pulmonary tuberculosis cases were defined as those with a positive culture for MTB in the respiratory specimen or those with positive clinical outcome (clinical and radiological improvement after six months of solely anti-TB treatment, as judged by three different chest physicians, who were not involved in this study, in a blinded review) [25]. Negative PTB patients were considered whose smear and culture for MTB were negative or who did not show chest radiographic changes after six months of follow-up. The gold-standard criteria for PTB final diagnosis included all PTB cases, regardless of whether they were confirmed by culture. Thus, the clinical outcome was considered the gold standard.

#### Performance and accuracy analysis

The epidemiological and laboratory data were entered into a computer database and analyzed with appropriate statistical software (SPSS version 16.0).

The endpoints were sensitivity (SE), specificity (SP), and the predictive negative and positive values (NPV, PPV) for detection in suspected PTB patients. For MTB DNA detection, the analysis of Detect-TB SE, SP, NPV and PPV were performed on a per-study-subject basis, using the diagnosis of PTB (defined above) as a reference standard.

Additionally, test performances of smear plus Detect-TB or culture as a diagnostic test were calculated using specific formulas: SE of smear plus Detect-TB or culture: SE smear + SE (Detect-TB or culture – (SE smear X SE (Detect-TB or culture), and predictive values (PV) for different prevalence rates, according to the literature [26].

#### Costs

The cost components for each procedure included the costs incurred by the patient, laboratory costs, drugs, consumables and equipment costs. The number and level of staff screening for TB in the hospital were considered to be the same for all strategies. Clinical, radiological and laboratory staff costs were calculated from the salary base of Rio Grande do Sul State of Brazil. For each procedure, the costs were attributed based on the procedure costs adopted by the Brazilian Public Health System. For Detect-TB, the capital costs included the cost of the thermocycler, microplate reader and centrifuge. Running costs (material costs used for each 1,000 tests evaluated) included all laboratory materials used in the procedures.

All costs were expressed in US$, using an exchange rate of US$ 1 = 3 R$ (Brazilian Real), by the procedure described by Scherer [20]. To estimate the values spent by the public health system of Brazil for the monitoring and control of TB in a hospital and ambulatory unit, we simulated two different scenarios: a) TB cases diagnosed in hospital wards (inpatients) and b) TB cases diagnosed in outpatient environment (outpatients). Therefore, to calculate the total treatment cost, the treatment of inpatients and outpatients were evaluated.

The number of days considered when calculating the costs related to the treatment of inpatients were considered the same as the days spent on each laboratory procedure. It was hypothesized that the time to detect MTB in sputum culture from patients with PTB would be a better indicator of the duration of hospitalization [27]. This cohort is the same as that in previous publication from our group [20].

#### Cost-effectiveness

A decision analytic model was developed using the Treeage Pro software®2009 to estimate the costs and cost-effectiveness of five routine diagnostic procedures for diagnosis of PTB using sputum specimens: a) Smear alone, b) Culture alone, c) Detect-TB alone, d) Smear plus culture and e) Smear plus Detect-TB.

The probabilities of events in the decision tree were derived from: a) Published studies and b) The SE and SP of the techniques. In this analysis, the tree was used to calculate the expected value per correctly diagnosed TB case. Table 1 shows the assumptions made in the construction of the decision tree. We evaluated the cost-effectiveness ratio of cost per correctly diagnosed TB case [28].

**Table 1:** **Model parameter value and ranges**

**Model assumptions**			
Discount rate (annual) (%)	0.03		
Time horizon	Lifetime		
Perspective	Societal		
**Study characteristics**			
Smear positive cases	26		
Culture positive cases	51		
Detect-TB positive cases	41		
Smear and culture positive cases	53		
Smear and Detect-TB positive cases	48		
**Variable**	**Probability Value**	**Range**	
Ambulatory TB prevalence	0.10	0.05	0.20
Hospitalization TB prevalence	0.30	0.25	0.35
Study TB prevalence	0.038	0.028	0.048
TB prevalence in RS (state in southern of Brazil)	0.047	0.037	0.057
SE smear	0.47	0.37	0.57
SE culture	0.93	0.83	0.99
SE Detect-TB	0.74	0.64	0.84
SE smear plus culture	0.96	0.86	0.99
SE smear plus Detect-TB	0.86	0.76	0.96

The SE analysis was performed to assess the effect of the various parameters (TB prevalence, SE, SP, and variable costs) on the outcomes.

## Results

A total of 390 spontaneous sputum samples were evaluated. Of these, 96 were excluded from analysis (5 showed culture contamination, 84 had an insufficient amount for molecular analysis, and 7 gave a reading in the gray zone).

### Comparative performances of smear, culture and detect-TB for PTB diagnosis

Of the 294 analyzed samples considering the clinical outcome as the gold standard, 55 samples were defined as TB cases and 239 non-TB cases. These results are displayed in Table 2. Between the 55 defined as TB cases, 26 (47%) showed positive results by smear, whereas 29 had false-negative results; 51 (93%) showed positive results by the culture, whereas 4 had false-negative results; 41 (74%) showed positive results by Detect-TB, whereas 14 (26%) had negative readings (false-negative). Among the 239 negative samples, 227 had negative results using Detect-TB, and 12 had positive readings (false positive). After this analysis, the SE and SP of Detect-TB were calculated at 74% (CI 95%: 68%–79%) and 95% (CI 95%: 92%–97%), respectively.

**Table 2:** **Performance of smear, culture and Detect-TB compared to the gold standard**

		N = 294	
		TB *N = 55*	Non-TB N = 239
**Smear**	**Positive**	26	0
	**Negative**	29	239
		**SE (%)**	**SP (%)**
		47	100
		(CI 95%: 41%–52%)	(CI 95%: 99%–100%)
**Culture**	**Positive**	51	0
	**Negative**	4	239
		**SE (%)**	**SP (%)**
		93	100
		(CI 95%: 89%–96%)	(CI 95%: 99%–100%)
**Detect-TB**	**Positive**	41	12
	**Negative**	14	227
		**SE (%)**	**SP (%)**
		74	95
		(CI 95%: 68%–79%)	(CI 95%: 92%–97%)
**Smear plus culture**			
		**SE (%)**	**SP (%)**
		96	100
		(CI 95%: 93%–98%)	(CI 95%: 99%–100%)
**Smear plus Detect-TB**			
		**SE (%)**	**SP (%)**
		86	95
		(CI 95%: 81%–90%)	(CI 95%: 92%–97%)

### Accuracy analysis and the areas of ROC curve of smear, culture, and detect-TB of PTB patients

**Figure 1:**
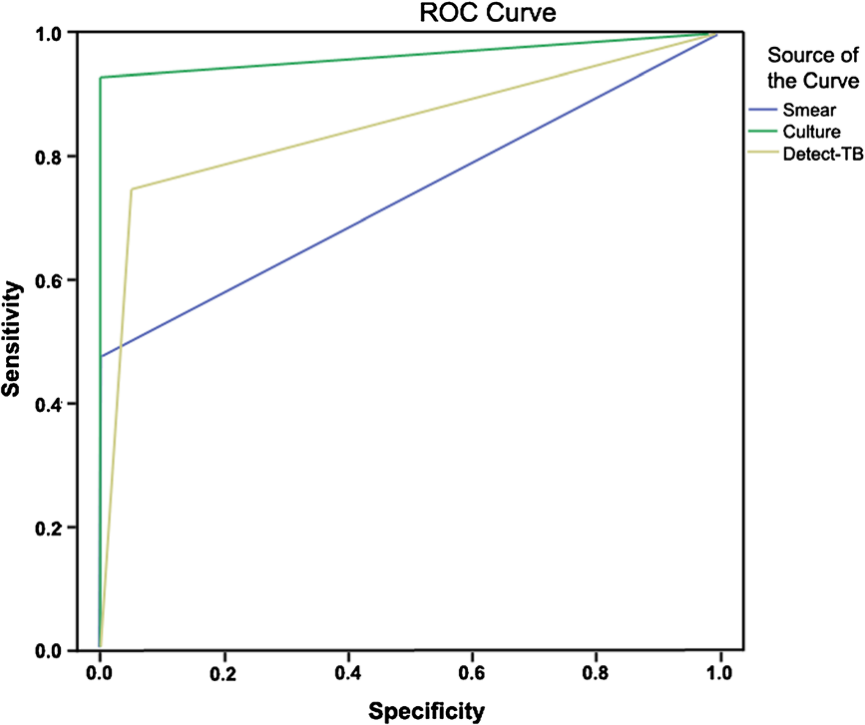
**Plot in the ROC space of accuracy estimates to each method and areas corresponding to 294 patients.** (blue: smear; green: culture; yellow: Detect-TB).

Among 294 patients with PTB suspects, the ROC analysis showed the areas of smear (0.736), culture (0.964) and Detect-TB (0.848), respectively (Figure 1).

### Costs

The median time to reveal the growth of MTB was 30 days (Interquartile Range [IQR] 30 to 45) for smear and culture, and the median time for detection of MTB by Detect-TB was 3.32 days (IQR 3.0 to 3.75) (p < 0.01). This value was used as the standard at which release from isolation could be permitted [29].

The times spent on each activity in the laboratory to provide results were assumed to be 2 days for smear plus Detect-TB and 30 days for smear plus culture. The numbers of days used to calculate costs was the same as those spent on each laboratory procedure. The numbers of days used to calculate patient travel costs were assumed to be 2 days for smear plus Detect-TB and 30 days for smear plus culture. The total treatment costs included the clinical officer (physician salary) and hospital costs; medication costs (based on US$ 0.22/pill, using 3 pills per day over 180 days); hospital room costs (US$ 4.16 per day); costs of clinical staff salary and clinical consultations (US$ 2.52 per patient); and costs of clinical nursing consultations (US$ 2.52 per patient). The outpatient treatment costs were calculated assuming the 6-month regimen recommended by the National TB program and included 6 smear tests, 6 chest radiographs, 6 nurse consultations and 2 physician consultations [30]. The inpatient treatment costs included the time of hospitalization, considered to be the same as the culture time (30 days); 4 smear tests; 4 chest radiographs and 30 nurse and physician consultations [30]. The staff salaries for the physician, nurse and radiologist were considered to be US$ 36,000 per year, and for the chest radiograph technician, the salary was US$ 12,000 per year. The monthly workdays were considered 20 days for all staff. The days of admission to the hospital were considered to be the same number of days used on each laboratory activity. All assessed costs reflect an estimate of the public health system of Brazil’s expenses regarding the monitoring and control of TB. The costs were expressed per 1,000 suspects, according to the specific bibliographic references for economic analyses, thus allowing the best decision for investment to be made [28]. In Table 3, the costs and assumptions made during construction of the decision tree are displayed.

**Table 3:** **Unit costs (in US$) of 1000 TB suspects screening, comparing smear plus culture and smear plus Detect-TB used in tree decision model**

	Smear plus culture	Smear plus Detect-TB
**Total cases TB**	53	48
**A health service costs (in US$)**		
**Laboratory costs** ^**a**^		
**Laboratory costs**	3,283	13,067
**Investment costs**	123	194
**Running costs**	12,333	12,833
**Treatment costs (outpatients)**	3,819	337
**Treatment costs (inpatients)**	3,690	328
**Treatment costs (outpatients/inpatients)**	7,508	665
**Diagnostic service costs per day** ^**a**^		
**Staff costs per activity-based costing**	1,158	77
**B1. Patient cost (outpatient)** ^**d**^		
**Travel**	24	2
**Food**	100	7
**Income loss**	175	12
**B2. Patient cost (inpatient)** ^**d**^		
**Travel**	0	0
**Food**	0	0
**Income loss**	175	12
**Total patient costs**	299,000	19,933
**Total health service costs**	1,180.748	103,926
**Total screening costs in 1000 TB cases**	1,479.748	123,859

^a^For each procedure, costs were attributed based on procedure costs of the Brazilian public health system (US$ 1.4 for smear and US$ 1.9 for culture) and from CDCT/FEPPS (US$ 11.7 for Detect-TB), assuming investment laboratory equipment for 5 years; ^b^Staff salary was considered; for laboratory technician, US$2,860 per year; for Laboratory technologist, US$6,400 per year. Staff costs in the laboratory were based on proportional days spent on each laboratory procedure; Staff salary was considered for clinical physician, nurse and radiologist; US$6,400 per year; for the X-RAY technician, salary was US$2,860 per year. ^c^The days of hospitalization were considered as the same as the days spent on each laboratory procedure. The time spent on each laboratory procedure until access to the result of the laboratory technique was assumed to be 2 days for smear plus Detect-TB and 30 days for smear plus culture. Total treatment included clinical officer and hospital costs, assuming US$ 0.22 cost per pill, using 3 pills for day, during 180 days; hospital room costs, US$ 4.16/day; costs of salary of staff clinical; clinical consultation cost, US$2.52 per patient; clinical nursing consultation, US$2.52 per patient. Assuming that during the treatment of hospitalized patients (4 months) 4 smear and 4 chest radiograph were performed, and during the treatment of outpatients (6 months) 6 smear and 6 chest radiograph were performed, following the Brazilian recommendations for treatment (Tuberculose 2004); ^d^Travel for smear strategies was considered as 30 days for smear plus culture strategy; and 2 days for smear plus Detect- TB. Food and income loss for smear strategies was considered as 30 days for smear plus culture strategy; and 2 days for smear plus Detect- TB. The health service costs analysis was based on processing 50 smear slides, 86 samples for each PCR (Detect- TB) and 14 cultures per day. Smear plus culture and Detect- TB were performed by two trained staff, respectively. Costs of chest physicians were considered the same for all strategies. Running costs were calculated from investments required to examine 1000 smears.

### Cost-effectiveness

#### Tree decision model

**Figure 2:**
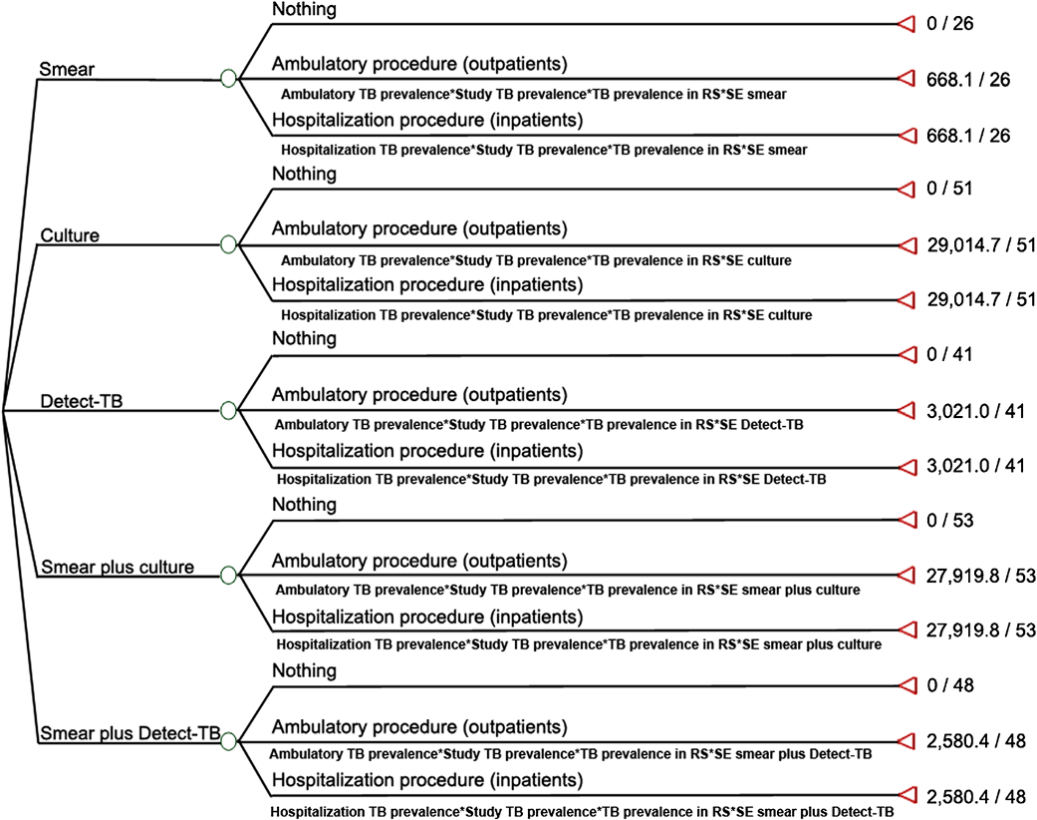
**Tree decision model.** Values = cost (US$) per detected case.

A decision analytic model (Table 1) was developed using the Treeage Pro software®2009 to estimate the cost-effectiveness of five diagnostic procedures for the diagnosis of PTB using sputum specimens: a) smear used alone; b) culture used alone; c) Detect-TB used alone; d) smear plus culture; and e) smear plus Detect-TB (Figure 2).

**Figure 3:**
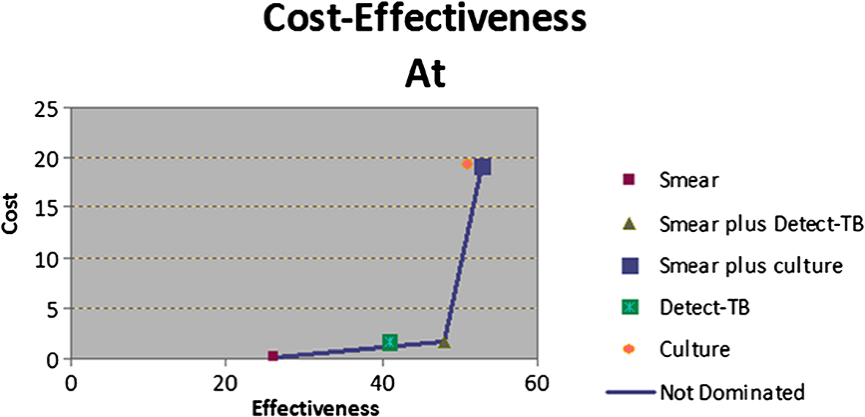
**Cost-effectiveness of five strategies for TB diagnosis.** (smear; culture; Detect-TB; smear plus Detect-TB; and smear plus culture). Cost-effectiveness = cost per detected case; cost = US$; effectiveness = detected case.

In the cost-effectiveness analysis shown in Figure 3, the smear alone strategy dominated, as it was both less expensive and less effective than the other strategies. Detect-TB and smear plus Detect-TB were less costly and also effective when compared with the other strategies.

In a cost-effectiveness analysis presented in Table 4, the ICER (Incremental Cost-Effectiveness Ratio) corresponds with the best value (i.e., lowest cost per unit of additional effectiveness). Smear plus Detect-TB showed the lower ICER (0.061865) when compared to the others strategies.

**Table 4:** **Cost-effectivenes of five strategies for TB diagnosis (smear; culture; Detect-TB; smear plus Detect-TB; and smear plus culture)**

Strategy	Cost per patient (US$)	Incremental cost (US$)	Effectiveness (Detected case)	C/E Ratio	ICER
Smear	0.224327		26	0.008628	
Culture	19.27714	2.12365	51	0.377983	(Dominated)
Detect-TB	1.59707	0.01171	41	0.038953	(Dominated)
Smear plus Detect-TB	1.585356	1.36103	48	0.033028	0.06186
Smear plus culture	17.15348	15.5681	53	0.323651	3.11362

## Discussion

The accuracy and cost-effectiveness of smear, culture and Detect-TB were evaluated in a prison population with a high prevalence of active PTB. We demonstrated that smear plus Detect-TB was the most cost-effective initial diagnostic test for active TB, indicating that Detect-TB has great potential to be implemented under routine diagnostic conditions. This is the first study in Brazil that assessed the cost-effectiveness of a molecular tool for TB diagnosis in a prison.

In PEJ, the TB prevalence was 72/1,900 (4,960/100,000) between 2007 and 2008, which is at least 100 times higher than the rate found in the civilian population [1]. In a Jail from Dourados city, Mato Grosso do Sul – Brazil, 249 of 1,261 inmates participated in the study for estimate active TB. The prevalence of the active disease was 400 / 100,000 [31]. In one prison and one jail in the city of Guarulhos, São Paulo – Brazil, a total of 2,435 inmates were screened for TB. The coefficient of prevalence of TB by the smear microscopy was 289,3/100,000 and by the culture was 1,079.7/100,000 [32]. Studies from Sub-Saharan African prisons suggest that 0.7% to 5.8% prisoners have undiagnosed active tuberculosis [33]. There is limited data on the prevalence of TB from prisons and these findings emphasize the increased risk of undetected TB in prisons.

A total of 294 patients with potential PTB were evaluated by smear, culture and Detect-TB. These results were compared to the clinical outcome, which was considered the gold standard. The SE and SP values were 47% and 100% for smear, 93% and 100% for culture, 74% and 95% for Detect-TB, 96% and 100% for smear plus culture, and 86% and 95%, for smear plus Detect-TB, respectively. The overall SE and SP of Detect-TB were similar to culture in the diagnosis of PTB.

In a previous study carried out by our group, the SE and SP of Detect-TB using spontaneous sputum were 85% and 98%, respectively [23]. A study performed on hospitalized patients in Uganda, in 2012, to diagnose the accuracy and impact of Xpert MTB/RIF among high-risk TB suspects, found a SE of 79% (CI 73%–84%), a SP of 96% (CI 92%–98%), a negative predictive value of 79% and a positive predictive value of 95% [34]. These data are in agreement with the findings obtained using Detect-TB. In another study on the diagnostic accuracy of Xpert MTB/RIF in HIV-infected patients, the SE was 91.7% (CI 64.6%–98.5%), the SP was 99.3% (CI 96.3%–99.9%), the NPV was 99.3%, and the PPV was 91.7%. This finding reinforces the fact that Detect-TB is comparable with commercially available kits. The SE, SP, NPV and PPV for Detect-TB are particularly interesting because the study population is from a prison, where the HIV positive rates are well-known to be 5- to 7-times higher than in the general population [35]. In the present study, it was not possible to assess the HIV rate. Any declared HIV status could have been included, but was not considered in this analysis. The use of molecular methods is particularly relevant in HIV-positive patients due to its paucibacillary nature and the difficulties in obtaining a satisfactory molecular test performance [36]. As the HIV status was obtained by declaration, sample stratification for statistical analyses was not performed. This can be considered an intrinsic study limitation. Furthermore, using smear plus Detect-TB as a TB diagnostic tool allowed for an earlier diagnostic assessment, many days in advance, than smear plus culture. Additionally, the difference between the accuracy of smear plus culture and smear plus Detect-TB was small (Table 2). The accuracy analysis was demonstrated by the ROC curve analysis, which mapped the areas of smear (0.736), culture (0.964) and Detect-TB (0.848), and showed that culture is the most accurate method for TB diagnosis when analyzed independently. Through the simulation of NPV and PPV considering different TB prevalence rates (Table 5), the smear plus Detect-TB NPV ranged from 83 to 99%, whereas the PPV ranged from 48 to 96%. Thus, high negative predictive values were observed with a TB prevalence of 4.9-20%, confirming that the strategy of using smear plus Detect-TB can be used to exclude TB cases in PEJ population. In addition, a high PPV for smear plus Detect-TB was observed, which was very similar to that of smear plus culture, reinforcing its good test performance, especially in high prevalence sites.

**Table 5:** **Simulation of positive and negative predictive values according to different TB prevalence rates**

Simulated prevalence rates		N = 294	
		TB N = 55	NTB N = 239
		**PPV (%)**	**NPV (%)**
**4.9%**	**Smear plus culture**	50	99
	**Smear plus Detect-TB**	48	99
		**PPV (%)**	**NPV (%)**
**10%**	**Smear plus culture**	68	99
	**Smear plus Detect-TB**	66	98
		**PPV (%)**	**NPV (%)**
**20%**	**Smear plus culture**	82	99
	**Smear plus Detect-TB**	81	97
		**PPV (%)**	**NPV (%)**
**30%**	**Smear plus culture**	89	98
	**Smear plus Detect-TB**	88	94
		**PPV (%)**	**NPV (%)**
**40%**	**Smear plus culture**	92	97
	**Smear plus Detect-TB**	92	92
		**PPV (%)**	**NPV (%)**
**60%**	**Smear plus culture**	96	94
	**Smear plus Detect-TB**	96	83

In this study, we demonstrated that smear plus Detect-TB provided more cost-effective results compared to routine methodology (smear plus culture). Though costs increase slightly, Detect-TB provides a more rapid diagnosis. It is already known that smear has a reduced SE, so it is necessary to use culture as a complementary method. This study evaluated the use of Detect-TB as a complementary method and showed great performance (Figure 3). Importantly, among the 24 smear-negative culture-positive samples, Detect-TB was able to identify 67% of them. This resulted in a gain using Detect-TB regardless of the use of culture, which translates into an increase in TB case detection, showing greater quickness and efficiency. This is a very important issue in developing countries, where TB is epidemic and scarce financial resources are available to public health [18],[37]. Early, accurate treatment becomes essential because this is the trigger point in the fight against resistance, and its spread in the general community, particularly in Brazil, where there are no visitor restrictions to prisoners and where weekly family visits are not uncommon. In these types of sites, there is no data on the performance and cost-effectiveness of molecular approaches. As for an intervention, the high cost of PCR itself is often used as an argument not to introduce it. However, its cost-effectiveness is a better argument for making such a decision [9]–[15]. In a cost-effectiveness analysis, sometimes a strategy can be eliminated based on its relative cost and effectiveness compared to another strategy. Using such an approach, an option is said to be dominated if both costs and effectiveness were more costly or less effective than a select strategy compared with the others, and in this case, the smear strategy is the most effective. According to the current data, the smear plus Detect-TB was the most cost-effective compared with the other strategies. The SE analysis indicated that significant factors contributed to the cost of strategies, time performance and the Detect-TB cost. In cost-effectiveness studies using indicators, such as the ICER, that describe the difference in cost, the difference in effectiveness between two scenarios is critical. The ICER is often reported as the cost per DALY (Disability-Adjusted Life Year Averted) and is always compared against a reference strategy [20]. In the present study, the ICER was reported as the cost per case detected and was US$ 0.061865 for the strategy smear plus Detect-TB. Evaluations of the cost-effectiveness of new diagnostic tests for TB in countries with resource constraints and high TB-HIV prevalence are still scarce, and much remains to be studied. In a recent study, the ICER of four hypothetical strategies for the diagnosis of TB (smear, a new test for the diagnosis, smear associated with the new test and smear associated with culture) were evaluated in Brazil, Kenya and South Africa. The smear was more cost-effective (Increment Cost per DALY), with US$ 86 [South Africa], US$ 131 [Brazil], and US$ 38 (Kenya], than the new hypothetical test, which showed a SE of 70% and a SP of 95%, and the costs of US$ 20 per test (US$ 198 [South Africa], US$ 275 [Brazil] and US$ 84 [Kenya]) [21].

The evaluation of the cost-effectiveness of molecular tests has emphasized the evaluation of commercial and in house testing. In a study conducted in 2005, the cost per correctly diagnosed case was US$ 41 for smear and US$ 67 for PCR (AMPLICOR). When treatment costs were included, PCR (AMPLICOR) was more cost-effective, US$ 382 versus US$ 412. In a recent study, the cost per correctly diagnosed case was US$ 50.773 for smear and culture and US$ 13.749 for smear and PCR dot-blot. The smear associated with PCR dot-blot was more cost-effective than smear-associated culture [20]. In the present study, the cost per correctly diagnosed case was US$ 29.014 for smear plus culture and US$ 3.021 for smear plus Detect-TB. Whereas the performance of a molecular test to diagnose PTB may be linked directly to the detection method [16],[17], in this study, the method of detection showed a reduction of 78% in the costs of the smear plus DETECT-TB strategy when compared to the use of smear associated with PCR-dot-blot in a previous study from our group.

The limitations of the study were: a) Pulmonary tuberculosis cases were defined as those with a positive culture for MTB in the respiratory specimen or those with clinical and radiological improvement after six months of solely anti-TB treatment; b) Stratification of different diagnostic strategy results by HIV status was not performed; c) Mortality was not measured for either strategy; d) For treatment cost analysis, costs related to the following were not included: I) The inadequate use of non anti-TB drugs; II) The adverse effects of the inadequate use of anti-TB drugs for non-TB subjects; III) The occurrence of drug-resistant TB (MDR-TB and non –MDR); IV) The delay in treatment and impairment of other physical conditions caused by adverse effects of drugs; V) The occurrence of new cases and previously treated cases; and VI) The incidence of male or female patients; e) Patient isolation use or contact investigation was not included in the analysis; f) In the cost-effectiveness model analysis were not used a Markov analysis because time was not determinant of outcome in our study. Our outcome was case-detected; g) We did not use the QALY as an indicator of effectiveness because we use TB cases diagnosed by the technique according established model; and h) These results are significant for evaluation during the current global phase of worldwide economic crisis, particularly in a country such as Brazil with a high prevalence of TB.

## Conclusions

Although some studies have demonstrated that PCR is more sensitive and specific, but also more costly, than other routine procedures [38], Detect-TB proved to be the most cost-effective procedure for diagnosis in this study, when used in combination with smear.

Diagnostic tests and procedures should be improved to increase the detection of TB cases. In addition to improving detection using procedures based on smear, alternative techniques should be considered. To allow rational decisions concerning the implementation of such techniques, cost-effectiveness studies are essential, including for prisons, which are crowded sites fraught with health care assessment problems. Such studies elucidate the composition of the different cost components, which should be evaluated from the perspective of the patient or health services.

## Authors’ information

KBS: Centro de Desenvolvimento Científico e Tecnológico (CDCT), Fundação Estadual de Produção e Pesquisa em Saúde (FEPPS), Porto Alegre, Brazil. LS: Universidade Luterana do Brasil (ULBRA), Canoas, Brazil. RBB: Centro de Desenvolvimento Científico e Tecnológico (CDCT), Fundação Estadual de Produção e Pesquisa em Saúde (FEPPS), Porto Alegre, Brazil. RES: Universidade Federal do Rio de Janeiro (UFRJ), Rio de Janeiro, Brazil. DK: Programa Nacional de Controle da Tuberculose - Secretaria de Vigilância em Saúde / Ministério da Saúde, Porto Alegre, Brazil. ERDC: Centro de Desenvolvimento Científico e Tecnológico (CDCT), Fundação Estadual de Produção e Pesquisa em Saúde (FEPPS), Porto Alegre, Brazil. MLRR: Centro de Desenvolvimento Científico e Tecnológico (CDCT), Fundação Estadual de Produção e Pesquisa em Saúde (FEPPS), Porto Alegre, Brazil.

## Authors’ original submitted files for images

Below are the links to the authors’ original submitted files for images. Authors’ original file for figure 1 Authors’ original file for figure 2 Authors’ original file for figure 3
