# Supplementary figures and images for: Smear plus Detect-TB for a sensitive diagnosis of pulmonary tuberculosis: a cost-effectiveness analysis in an incarcerated population
Source: BMC Infect Dis. 2014 Dec 16;14:678. doi: 10.1186/s12879-014-0678-x (PMC4299548; doi:10.1186/s12879-014-0678-x)

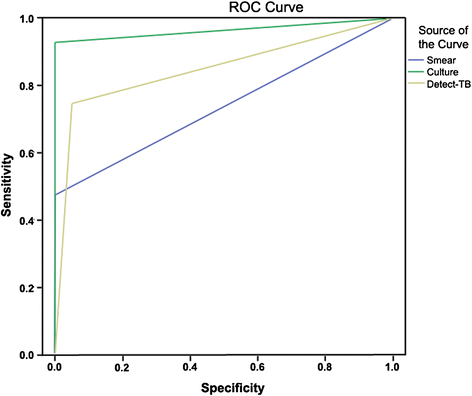

Supplement: Supplementary file 1 — Authors’ original file for figure 1 [file 12879_2014_678_MOESM1_ESM.gif]

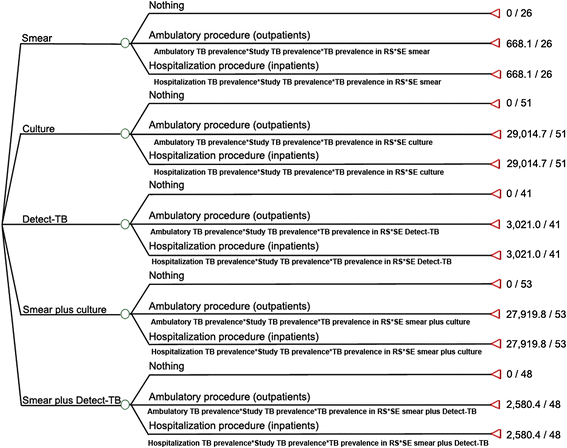

Supplement: Supplementary file 2 — Authors’ original file for figure 2 [file 12879_2014_678_MOESM2_ESM.gif]

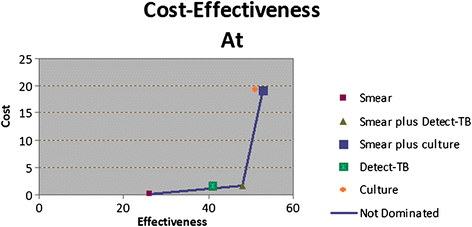

Supplement: Supplementary file 3 — Authors’ original file for figure 3 [file 12879_2014_678_MOESM3_ESM.gif]
